# Lymphatic malformations: mechanistic insights and evolving therapeutic frontiers

**DOI:** 10.1172/JCI172844

**Published:** 2024-03-15

**Authors:** Milena Petkova, Ingvar Ferby, Taija Mäkinen

**Affiliations:** 1 Department of Immunology, Genetics and Pathology, Uppsala University, Uppsala, Sweden.; 2 Wihuri Research Institute, Biomedicum Helsinki, Helsinki, Finland.; 3 University of Helsinki, Helsinki, Finland.

## Abstract

The lymphatic vascular system is gaining recognition for its multifaceted role and broad pathological significance. Once perceived as a mere conduit for interstitial fluid and immune cell transport, recent research has unveiled its active involvement in critical physiological processes and common diseases, including inflammation, autoimmune diseases, and atherosclerosis. Consequently, abnormal development or functionality of lymphatic vessels can result in serious health complications. Here, we discuss lymphatic malformations (LMs), which are localized lesions that manifest as fluid-filled cysts or extensive infiltrative lymphatic vessel overgrowth, often associated with debilitating, even life-threatening, consequences. Genetic causes of LMs have been uncovered, and several promising drug-based therapies are currently under investigation and will be discussed.

## The lymphatic vasculature: guardian of tissue homeostasis

In recent years, research has greatly advanced our knowledge about lymphatic vessels and their roles in a broad range of physiological and pathological conditions. The major function of the lymphatic system is to maintain tissue fluid homeostasis by absorbing interstitial fluid and returning it to the blood circulation (1, 2). It also provides a trafficking route for immune cells and peripheral antigens to lymph nodes and is responsible for the absorption of dietary fat. Recent findings have challenged the prevailing perception of the lymphatic system as a mere conduit for fluids and immune cells. For example, lymphatic endothelial cells (LECs) within the lymph node play a critical role in the modulation of immune responses by controlling the traffic of immune cells as well as influencing immune cell activation and tolerance induction (3). Furthermore, paracrine signaling between LECs and other cell types has been reported to control growth and regeneration of various tissues, including the heart and intestine (4–7), and contribute to metabolic homeostasis (8).

Morphologically, the lymphatic system comprises a vascular network consisting of blind-ended lymphatic capillaries, also known as initial lymphatics, which drain into a network of valved collecting vessels and larger ducts that ultimately return the fluid to the venous blood. The two distinct types of lymphatic vessels have unique structural and molecular features that align with their specialized functions (9). The capillary LECs facilitate fluid uptake by permeable button-like intercellular junctions, and they lack a continuous basement membrane (10). In contrast, collecting vessels are adapted to prevent leakage of lymph due to their tighter zipper-like cell-cell junctions and a continuous basement membrane. Additionally, smooth muscle cells surrounding the collecting vessels have the capacity to contract, working alongside with luminal valves to propel lymph fluid forward (1, 2).

Abnormalities in the growth, structure, or function of the lymphatic vessels resulting from genetic defects or as a consequence of damage, e.g., caused by surgery, trauma, or radiation, lead to various health problems (1, 11). Technological advances in next-generation sequencing have allowed the identification of genetic causes of a variety of lymphatic anomalies (12–14). This molecular understanding has not only provided important insight into the regulation of lymphatic vessel growth and diseases, but also opened up new possibilities for therapeutic targeting. In addition, single-cell transcriptomics has begun to reveal a striking molecular heterogeneity within the lymphatic endothelium across different vessel types and organs (15). We are just beginning to uncover the likely organ-specific roles of lymphatic vessels, and a better understanding of those roles is likely crucial for insight into a variety of pathologies, such as lymphedema, inflammation, obesity, and atherosclerosis.

In this Review, we will focus on a form of lymphatic anomaly, called cystic lymphatic malformation (LM), which is a localized lesion presenting with lymphatic vessel overgrowth (13). For a comprehensive review of other types of lymphatic anomalies, including lymphedema and complex lymphatic anomalies (CLAs), we refer to recent publications (13, 14, 16–19).

## Clinical characteristics of LMs

**Figure 1:**
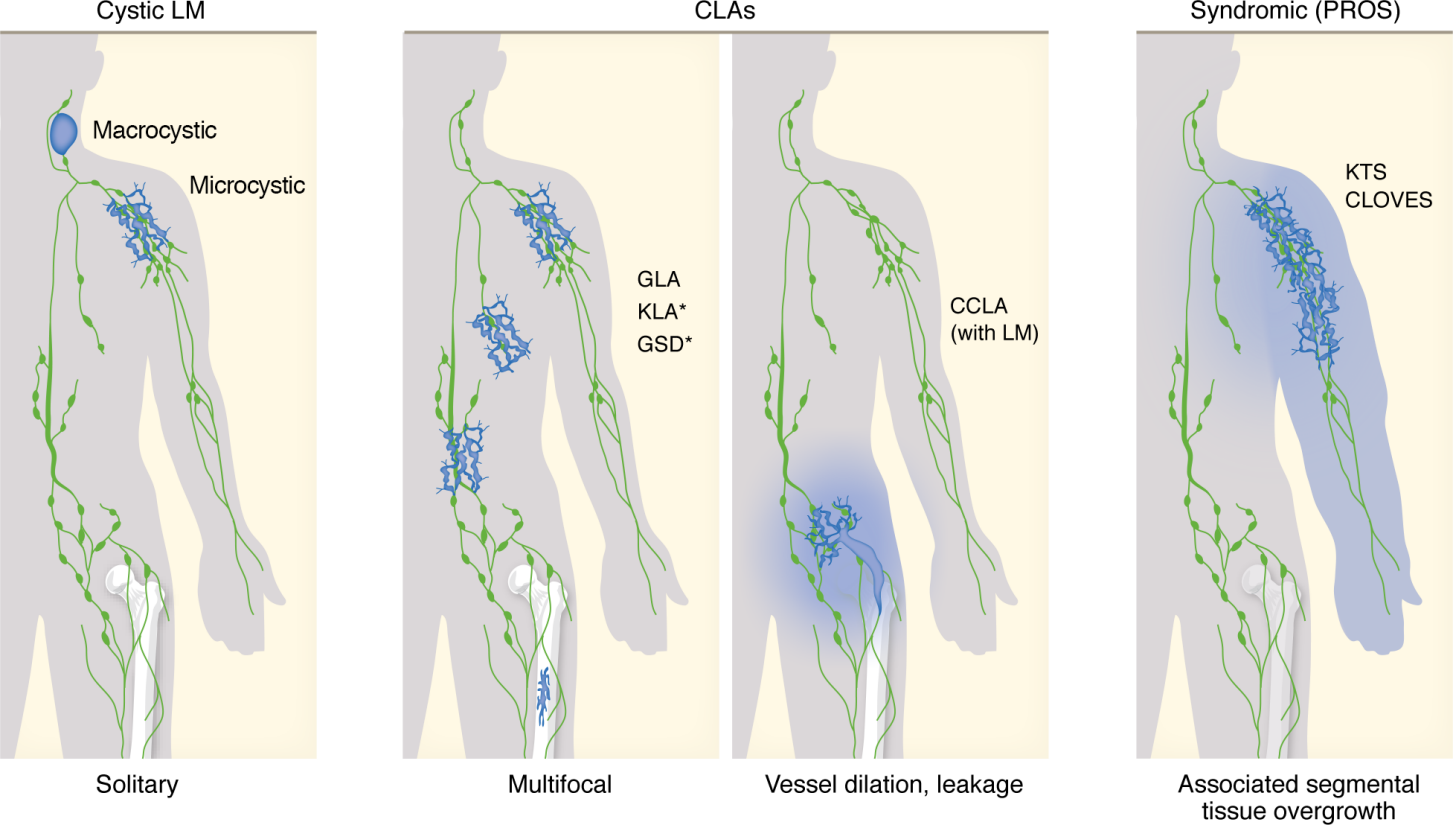
Features of genetic lymphatic anomalies associated with lymphatic vessel overgrowth. Schematic representation of the main clinical features of lymphatic anomalies characterized by lymphatic overgrowth (indicated in blue): cystic LMs, complex lymphatic anomalies (CLAs), and LMs associated with segmental overgrowth syndromes (PROS). The following definitions are based on the classification for vascular anomalies proposed by the International Society for the Study of Vascular Anomalies (ISSVA): LM, lymphatic malformation; GLA, generalized lymphatic anomaly; KLA, Kaposiform lymphangiomatosis; GSD, Gorham-Stout disease; CCLA, central conducting lymphatic anomaly; PROS, PIK3CA-related overgrowth spectrum; KTS, Klippel-Trenaunay syndrome; CLOVES, congenital lipomatous overgrowth, vascular malformations, epidermal nevi, and scoliosis/skeletal/spinal anomalies. KLA and GSD are characterized by bone lesions (as indicated with asterisks), and CCLA is associated with dilation of and leakage from central lymphatic vessels.

LMs are rare congenital abnormalities of the lymphatic system, with an estimated occurrence of approximately 1:4,000 live births. In rare cases, LMs have been reported to manifest in adulthood (13). These malformations appear as solitary lesions of variable size, which typically grow proportionally with the size of the body. LMs are commonly located in the head and neck region, as well as in the abdominal and thoracic areas, but they can affect the skin and soft tissues in virtually any part of the body (Figure 1) (13, 17).

LM lesions are classified based on their overall morphology into two types: macrocystic LM (previously known as cystic hygroma) and microcystic LM (previously known as lymphangioma) (Figure 1) (17). The distinction between the two types is not always evident, and in some cases, an individual patient may present with both types of lesions. Their location, size, and morphology as well as their impact on the neighboring tissues cause various symptoms and disease effects in patients. Microcystic LMs, due to their tissue-infiltrating nature, are generally associated with the most severe complications, which include acute pain, organ dysfunction, and, in some cases, even mortality, e.g., due to airway obstruction. Even small LMs can have severe clinical effects when exposed to additional factors such as acute infection, trauma, or hormonal changes, which may trigger rapid growth of the lesions (13).

Lymphatic lesions with similar characteristics can also manifest in a multifocal fashion, affecting several tissues. These conditions include generalized lymphatic anomaly (GLA), Gorham-Stout disease (GSD), and kaposiform lymphangiomatosis (KLA), which are commonly referred to as CLAs (Figure 1) (17–19). LMs can occur alongside other types of vascular overgrowth, such as venous malformations or capillary malformations, resulting in mixed vascular lesions (13). LMs are also frequently observed as part of overgrowth syndromes collectively known as PIK3CA-related overgrowth spectrum (PROS) (Figure 1), which include conditions like Klippel-Trenaunay syndrome and congenital lipomatous overgrowth, vascular malformations, epidermal nevi and scoliosis/skeletal/spinal anomalies (20, 21). Notably, some of the most aggressive vascular anomalies tend to have a lymphatic component (19).

While the anomalies discussed above are primarily somatic diseases, there are also several hereditary conditions associated with lymphatic defects. For example, hereditary lymphedema, typically resulting from lymphatic hypoplasia or dysfunction, differs clinically and genetically from LM (16). Nonetheless, recent studies reported the co-occurrence of lymphedema and microcystic LM in a patient with a type of CLA known as central conducting lymphatic anomaly (CCLA) (22) (Figure 1) as well as in another patient with Parkes Weber syndrome (PWS) (23). The latter condition is characterized by arteriovenous and capillary malformations, along with limb overgrowth, resulting from germline *RASA1* and *EPHB4* mutations (24, 25). CCLA can be caused by both germline and somatic mutations in various genes, which primarily result in elevation of the RAS/MAPK pathway, or chromosomal abnormalities, which result in a heterogenous lymphatic disorder that affects the large lymphatic collectors (14, 26, 27). Notably, in both cases of co-occurring lymphedema and LM associated with CCLA or PWS, the disease was attributed to a somatic mutation in *KRAS* (22, 23). These instances underscore the challenges in classifying lymphatic anomalies, given their heterogeneity and overlapping symptoms. This emphasizes the need to consider not only clinical manifestations, but also the genetic causes, which have been rapidly unveiled during the past decade (19).

## Genetics and diagnosis of LMs

**Figure 2:**
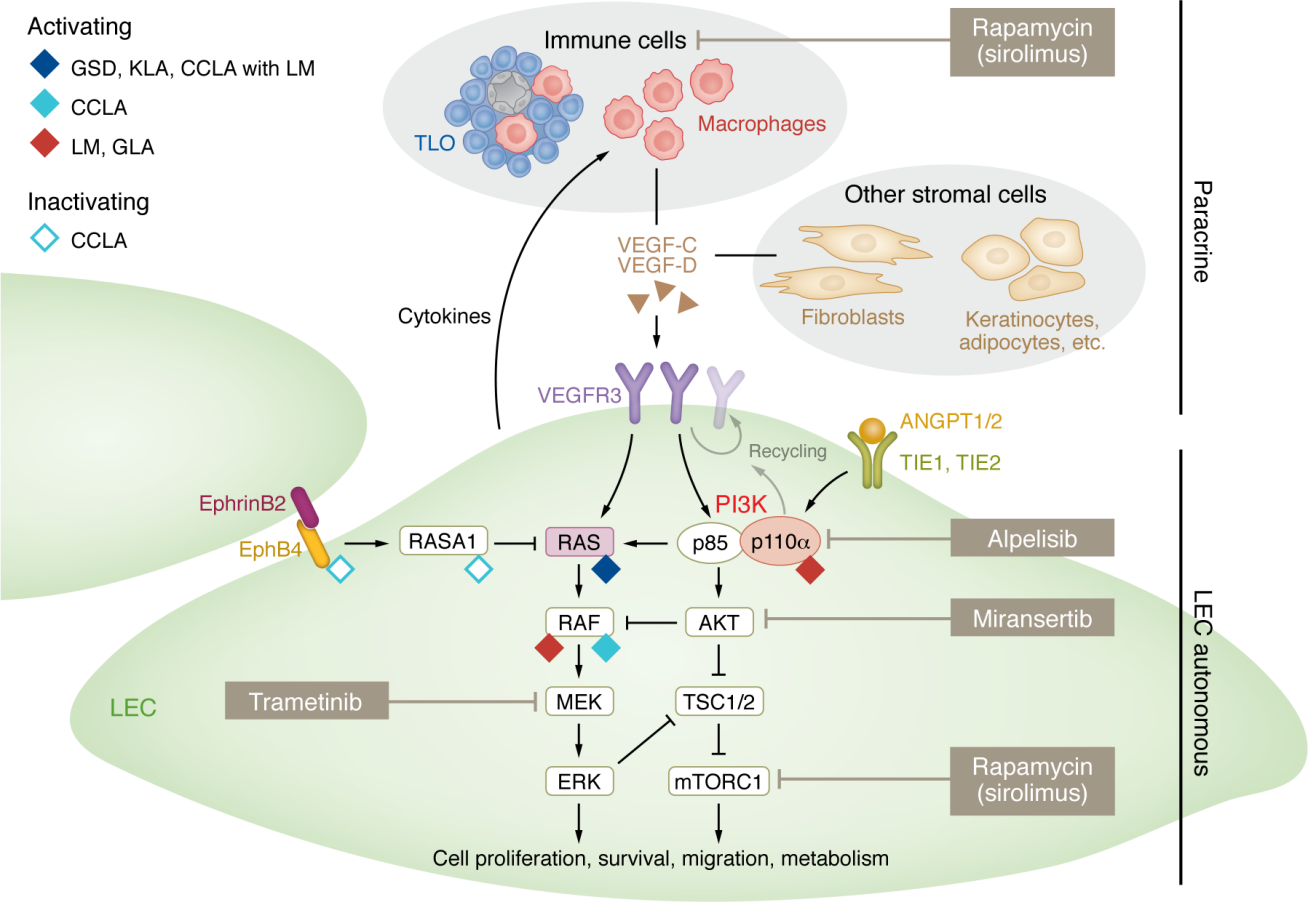
Signaling pathways and therapeutic opportunities for vascular anomalies associated with lymphatic overgrowth. Schematic overview of the RAS/MAPK and PI3K/AKT LEC signal transduction pathways involved in LM and CLAs (GSD, KLA, CCLA, and GLA). Activating and inactivating mutations in disease-causative proteins have been indicated. Lines ending with arrowheads indicate positive regulations, and lines ending with bars indicate inhibitory regulations of substrate proteins. A somatic activating *PIK3CA* (encoding PI3Kα) mutation in LECs leads to a cell-autonomous increase in PI3K/AKT signaling as well as LEC proliferation and migration and can be targeted with PI3K pathway inhibitors, including alpelisib (targeting PI3Kα, currently in clinical trials for LM treatment), miransertib (targeting AKT), and rapamycin (targeting mTOR, also currently in trials for LM treatment). RAS/MAPK pathway activating mutations mainly found in GSD, KLA, and CCLA can be targeted with the MEK inhibitor trametinib (blue). Paracrine signaling between mutant LECs and stromal cells contributes to pathological vascular growth, potentially offering additional therapeutic options. For example, infiltration of VEGF-C–producing macrophages, which is actively driven by LECs, promotes pathological *Pik3ca*-driven lymphatic overgrowth that can be inhibited by VEGF-C blockade or antiinflammatory COX2 inhibition in mice (51). Antiinflammatory properties of rapamycin may contribute to its beneficial effects in limiting LM growth. LM, lymphatic malformation; GLA, generalized lymphatic anomaly; KLA, Kaposiform lymphangiomatosis; GSD, Gorham-Stout disease; CCLA, central conducting lymphatic anomaly; TLO, tertiary lymphoid organ composed of lymphocytes (blue), macrophages (red), and specialized high endothelial venules (gray).

The majority of isolated cystic LMs arise from somatic activating mutations in *PIK3CA* (28, 29), the gene encoding the catalytic subunit of the lipid kinase PI3Kα, which regulates diverse cellular processes and is essential for normal vascular development (Figure 2) (20). LM-causative mutations are identical to those found in GLA (30), KLA (31), venous malformations (32–34), PROS, and cancer (21). However, the frequencies of the specific mutations differ in the different disease conditions (35, 36) and may lead to a different degree of activation of downstream signaling via the serine-threonine protein kinase AKT and mTORC1 (36). Most *PIK3CA* mutations associated with LM and cancer cluster at three specific “hot spots” located in the helical domain (E542, E545) and the kinase domain (H1047) of PI3Kα (20, 21). In contrast, non–hot spot mutations were reported to be more common in the syndromic form of lymphatic anomalies associated with PROS than in isolated LMs (35), they were also found in some cases of CLA (31). Within LM, non–hot spot mutations show a higher prevalence in macrocystic lesions compared with microcystic lesions (36). In addition, a recent study reported significantly higher genotype-adjusted variant allele frequency of *PIK3CA* hot spot mutations in clinically more severe orofacial microcystic lesions, compared with neck/body-located macrocystic ones (36).

Recently, somatic activating mutation in *BRAF* was identified in three patients with isolated LM, which was histologically indistinguishable from *PIK3CA*-related macrocystic LM (37). Although a larger cohort is needed to validate conclusions, *BRAF*-related LM appeared to present a milder phenotype compared with LM with *PIK3CA* mutations. As discussed above, activating *KRAS* mutations were found in patients with CCLA and PWS associated with lymphedema and microcystic LM (22, 23). These findings indicate the involvement of the RAS/MAPK pathway, previously primarily associated with CLAs (38–42), in LM formation (Figure 2), highlighting the importance of genetic diagnosis before initiating medical therapy. Several additional candidate genes associated with LM have been identified in recent years, but their functional validation and confirmation in additional cases will be important for establishment of their disease-causing roles. Among the identified genetic defects is an activating mutation found in *PIK3CD* (43), which encodes PI3Kδ.

The diagnosis of LM relies on a combination of clinical evaluation and medical imaging. In particular, T2-weighted MRI is an important tool for the visualization of the extent of the malformation, especially if the lesion is located deep within the tissue (13). Detecting mutations present in LECs as low-level mosaicism in tissue obtained from a biopsy or resected during surgery has been challenging in genetic testing. Given that the majority of LM cases are caused by a *PIK3CA* mutation, PIK3CA-targeted pharmacotherapy is sometimes considered following diagnosis, even prior to genetic testing. However, recent technological advancements in next-generation sequencing and digital-droplet PCR have significantly increased the sensitivity of technology used to detect somatic mutations. The increased sensitivity has enabled detection of mutations using amplified cell-free DNA from both plasma and cyst fluid (44, 45), thus providing a minimally invasive solution for diagnostics. Another approach to enhance mutation detection is to isolate LECs from lymphatic fluid (45) or a biopsy of the affected tissue, e.g., by flow cytometry (46), or to use a recently developed method based on in vivo “lumen digestion” to detach and recover mutant cells from the lesion without disruption of the normal surrounding tissue (47). These advancements will contribute to more accurate and accessible genetic testing for LMs and other somatic diseases.

## Oncogenic pathway activation in LECs driving LM growth

The cellular mechanisms that underlie LM growth have been studied in patient-derived LECs (29, 48). In addition, genetic mouse models expressing the frequently occurring, LM-causative hot spot mutation *Pik3ca^H1047R^* (30, 49) or a constitutively active PI3Kα chimera (50) specifically in LECs were generated. In line with the known functions of PI3K, *Pik3ca^H1047R^*-expressing LECs showed increased basal AKT activity as well as increased proliferation and migration both in vitro and in mice (29, 48, 49, 51) (Figure 2). Notably, LECs from mutant mice (49) and human lesions (48, 52) exhibited increased levels of VEGFR3 and neuropilin 2, which serve as receptors for the major prolymphangiogenic growth factor VEGF-C. Stimulation of *Pik3ca^H1047R^*-expressing LECs with VEGF-C led to a further aberrant increase in AKT activity in vitro, while inhibiting VEGF-C was found to effectively inhibit *Pik3ca*-driven lymphatic hypersprouting of microcystic LMs in mice (49). Interestingly, LM lesions in mice displayed a prominent presence of macrophages producing VEGF-C (51); this appears to constitute an important component of progressive LM growth, at least in mice, as will be further discussed below.

Due to the availability and success of the mTOR inhibitor rapamycin in the treatment of LM and other vascular malformations, there has been a strong focus on the role of AKT/mTOR signaling in LM pathogenesis. However, it should be noted that AKT has many additional targets that may also play a role and offer targets for improved therapy. Given the observed requirement of VEGF-C/VEGFR3 signaling for LM growth, other VEGFR3 downstream pathways, such as the RAS/MAPK pathway, are likely also to be of importance. Phosphorylation of both AKT1 and ERK1/2 was indeed observed in biopsies of patients with LM (53, 54). Regulators of endosomal recycling that sustain VEGFR3 levels at the plasma membrane may also be of therapeutic relevance. This is inferred by the recent finding that TIE receptor signaling is required for VEGF-C–induced lymphangiogenesis by promoting PI3K-dependent recycling of VEGFR3 to the plasma membrane (55) (Figure 2).

The cellular effects of direct genetic activation of the Ras/MAPK pathway in LM and CLAs have also been experimentally modeled. LECs isolated from a patient with advanced KLA carrying an activating *NRAS* mutation showed increased sprouting and enhanced activity of both MAPK and AKT (56). Similarly, CCLA-associated *KRAS* and *ARAF* variants induced abnormal cell morphology and sprouting, along with increased MAPK activation, in cultured LECs and led to abnormal lymphatic development in zebrafish (22, 57). In mice, hyperactive KRAS signaling induced ectopic lymphatic vessels in bone and defects in large collecting vessels and their valves, reminiscent of GSD and CCLA, respectively (39). Importantly, the cellular defects in these models were inhibited by the MEK inhibitor trametinib (22, 39, 56, 57), providing a basis for therapeutic targeting of the Ras/MAPK pathway for the treatment of these diseases.

## Current therapeutic opportunities for LM

Patients with LM have until recently been treated solely by nonpharmacological approaches, such as surgical resection, sclerotherapy, and laser therapy to provide local control and symptomatic relief, but these treatments are generally only effective on well-localized micro- and macrocystic LMs. The identification of oncogenic mutations that result in activation of the well-known PI3K/AKT/mTOR and RAS/MAPK pathways has enabled exploration of drug-based therapeutic interventions using small-molecule inhibitors that were developed for cancer treatment (Figure 2). In particular, inhibitors of mTOR and PI3Kα have shown efficacy in preventing *PIK3CA*-driven LM and survival in mouse models of LM (30, 49, 50, 58), and several clinical trials targeting vascular anomalies are ongoing (Table 1). Some trials report promising effects in the treatment of lymphatic anomalies and will be discussed here.

The mTOR inhibitor rapamycin (trade name sirolimus) is a commonly used drug in clinical practice because of its immunosuppressive properties. Several phase I and II clinical trials (Palvella Therapeutics, NCT05050149; Lille University Hospital, NCT03243019) as well as a large multicenter phase III clinical trial (VASE, EudraCT no. 2015-001703-32; NCT02638389) have evaluated or are currently evaluating rapamycin efficacy and safety in pediatric and adult patients with vascular malformations, including LMs (Table 1). A review of 16 early studies, mostly consisting of case studies, including a total of 52 patients treated with sirolimus for microcystic LM, showed promising results. Clinically meaningful long-term improvement (up to 3 years) was noted in 92% (46 of 50) of the participants. Sirolimus yielded improvements in manifestations such as lymphatic leakage, bleeding, vesicle bulk, pain, and skin discoloration. Some participants experienced a rapid onset of effect within 2 weeks (59). Preliminary analysis of the larger phase III VASE trial on the first 101 patients with at least 6 months of treatment showed that 87% experienced functional improvement and less pain (60), although regression of overgrowth is limited. Earlier treatment, prior to active overgrowth, is likely to be more effective. Notably, a recent case report describes prenatal treatment with rapamycin of a fetus with a cervicofacial fetal LM with encouraging long-term outcome and limited side effects (61). In addition to inhibiting the AKT/mTOR pathway in endothelial cells that express the mutant *PIK3CA*, rapamycin exerts immunosuppressive effects through regulation of T cell function (62). These antiinflammatory functions may contribute to the beneficial effects of rapamycin in limiting LM growth.

The PI3K inhibitor alpelisib (BYL719) selectively inhibits the PI3Kα subunit (63) and was originally FDA approved for treatment of *PIK3CA*-mutated breast cancer. In 2022, alpelisib was approved by the FDA for patients with severe PROS requiring systemic treatment (Novartis; Vijoice, NCT04085653). Alpelisib has also been administered in a pilot study on LMs. Treatment of 6 patients, including 3 children, with LM for 6 months showed a 48% decrease in the size of lymphatic lesions (50), thus meriting further exploration of the use of alpelisib on LMs. Alpelisib is currently in phase II/III clinical study for pediatric and adult patients with *PIK3CA*-related LM (Novartis NCT05948943) (Table 1).

Other molecular treatments that target the PI3K pathway include the AKT inhibitor miransertib (Figure 2), which is currently in a phase II clinical trial for patients with PROS or Proteus syndrome (Merck Sharp & Dohme, NCT04980872). Miransertib is shown to inhibit PI3K signaling and decrease cell viability in patient-derived *PIK3CA*-mutant cells from venous malformations (64), but no trials specifically addressing the effect on LM are currently ongoing.

Inhibition of the RAS/MAPK pathway through MEK inhibition (Figure 2) has shown significant improvement in the lymphatic phenotype for patients with KLA (56, 65), CCLA (22, 57), and Noonan syndrome (66). Despite heterogeneity in both phenotype and genetics among the treated patients carrying gain-of-function mutations in positive regulators (*NRAS, ARAF, SOS1*) (56, 57, 66) or loss-of-function mutations in a negative regulator (*CBL*) (65) of the RAS/MAPK pathway, the observed clinical effects suggest MEK inhibitors as promising candidates for evaluation in clinical trials for patients with cystic LMs, in particular those associated with CLAs and unresponsive to sirolimus therapy. Currently, two MEK inhibitors, mirdametinib and trametinib, are undergoing clinical trials for fast-flow vascular malformations that are also associated with defects in RAS/MAPK pathway genes (NCT05983159, NCT06098872).

Additional medical approaches include sclerotherapy agents picibanil (OK-432) and TARA-002, which are in phase II and III clinical trials for LM (University of Iowa, NCT00010452; Protara Therapeutics, NCT05871970). These consist of a lyophilized strain of inactivated *Streptococcus pyogenes* bacteria, devoid of infectious properties, and are administered through intralesional injection. Originally developed as a stimulant for immune cytotoxicity against cancer (67), this treatment may also promote fibrotic changes within the LM lesion (68). Another widely used sclerotherapy agent is bleomycin, which is extracted from *Streptomyces verticillus*, that induces cell apoptosis. Additional sclerosing agents include ethanol, doxycycline, hypertonic glucose solution, and corticosteroids. There are complications associated with the use of each of these agents (69). However, OK-432 and bleomycin have shown highly beneficial results in the treatment of macrocystic LMs (67) and LMs of the head and neck (70), respectively, and continue to be the most commonly used treatments in clinical practice.

## Emerging role of stromal interactions in LM pathogenesis

**Figure 3:**
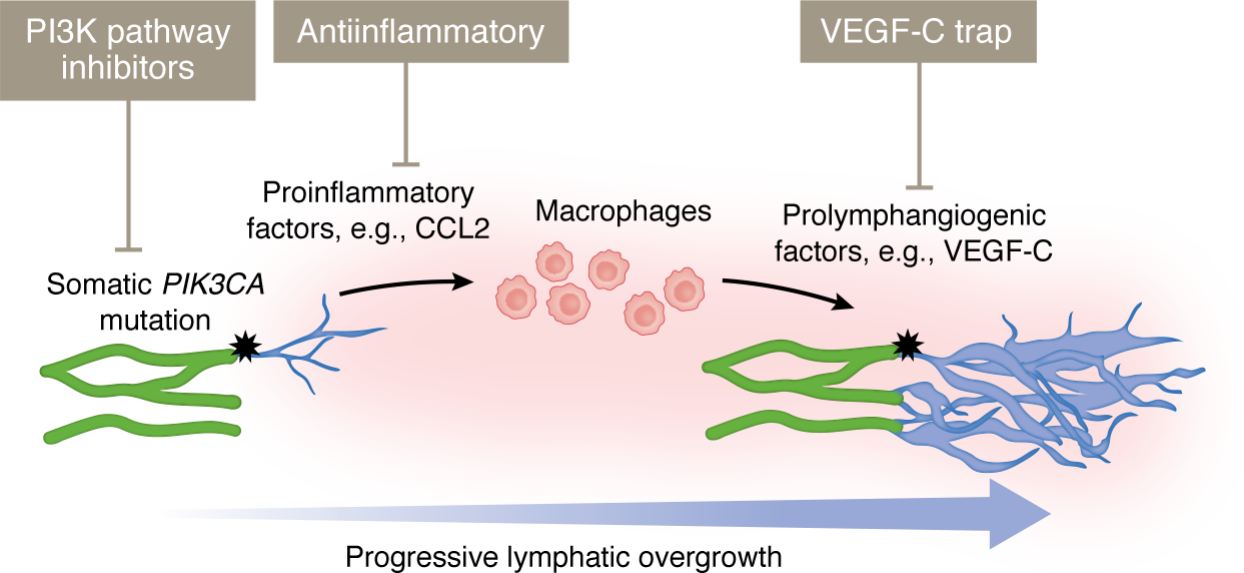
Proinflammatory paracrine signaling as a potential target for the treatment of *Pik3ca^H1047R^*-driven microcystic LM. In a mouse model of LM, expression of oncogenic *Pik3ca^H1047R^* (black stars) in LECs (green vessels) leads to lymphatic vessel overgrowth (blue vessels). This overgrowth is associated with a proinflammatory phenotype of LECs, promoting the recruitment of macrophages that, in turn, produce prolymphangiogenic VEGF-C and drive progressive vessel growth. In addition to PI3K pathway inhibitors, the use of antiinflammatory therapy, such as COX2 inhibitor celecoxib, and VEGF-C blockade has proven effective in limiting *Pik3ca*-driven lymphatic overgrowth in mice (51).

Observations from murine models of LM suggest that LEC-autonomous activation of PI3K signaling is not sufficient to promote progressive LM growth in the absence of VEGF-C signaling, suggesting an opportunity for therapeutic targeting. In normal tissues, *VEGFC* is mainly expressed in arterial endothelial cells and fibroblasts, but it is also expressed by multiple other cell types such as smooth muscle cells, keratinocytes, adipocytes, and immune cells (ref. 71; Human Protein Atlas, http://www.proteinatlas.org). In particular, in pathological conditions such as inflammation and cancer, the role of macrophages as a source of VEGF-C is well established (72, 73). The presence of immune cell infiltrate, composed predominantly of macrophages, has been observed in LM, both in murine models (51) and human lesions (74). This recruitment is mediated through production of proinflammatory chemokines by a newly discovered subtype of LECs expressing pentraxin 3 (*Ptx3*), termed immune-interacting LECs (iLECs), located at lymphatic capillary terminals, that was found to increase in number in the murine LM lesions (51) (Figure 2). Inhibition of macrophage recruitment, macrophage depletion, and antiinflammatory COX-2 inhibition limited *Pik3ca*-driven lymphangiogenesis in this experimental model of microcystic LM (51). Targeting the paracrine crosstalk between iLECs and macrophages may thus offer a novel therapeutic opportunity, given the accessibility and safety of antiinflammatory drugs (Figure 3).

In addition to macrophages, iLECs may interact with other immune cell types, such as T cells, which have been found to accumulate and organize into tertiary lymphoid organs (TLOs) within LM lesions in patients (75) (Figure 2). TLOs are lymph node–like aggregates of lymphocytes and antigen-presenting cells, frequently found at sites of nonresolved chronic inflammation (76). They have been associated with favorable prognosis in some cancers but worse disease progression during chronic inflammation (76, 77); however, their role in LM pathology remains to be clarified. Defective lymph outflow observed in LM has been proposed to contribute to immune cell retention and TLO formation (78). TLO formation can also be actively promoted by proinflammatory macrophages recruited to both mouse (51) and human LM (74), which are key inducers of TLOs in other conditions (79). Subsequently, a feed-forward mechanism appears plausible whereby iLEC-recruited macrophages facilitate the formation of TLOs (51). These TLOs, in turn, contribute to the increased production of VEGF-C, and other proinflammatory factors within LM lesions further promoting their growth. This is supported by data showing that TLO formation is associated with inflammation-induced neolymphangiogenesis both in mice (80) and humans (81). Notably, during infection, there is an increase in TLO numbers and the abundance of macrophages within them (74), which may contribute to the rapid growth of microcystic LMs observed as a result of infection in some patients (82). Taken together, inflammation emerges as an important promoter of LM growth, raising the possibility that antiinflammatory treatments hold promise as an effective therapeutic strategy for patients with LM.

Paracrine crosstalk between fibroblasts and blood ECs was recently shown to contribute to the growth of *PIK3CA*-driven venous malformations with a fibrous component (83). In this communication, mutant ECs secrete TGF-α, which stimulates VEGF production in fibroblasts and thereby promotes malformation growth. Fibroblasts can also function as paracrine regulators of lymphangiogenesis by producing VEGF-C (84) as well as the second VEGFR3 ligand, VEGF-D (85). Fibroblast-derived amphiregulin, which is a ligand for the EGF receptor, was suggested to promote lymphatic cyst formation in mice and in patients with macrocystic LM (85).

Organ-specific differences in the stromal cell composition and their responses to LEC-derived signals may partly explain tissue-specific differences in disease manifestation. Such variations in the microenvironment could also account for the observed differences in lesion manifestation depending on the timing of *Pik3ca^H1047R^* expression, with early embryonic transgene induction leading to macrocystic LM and later activation resulting in microcystic LM in mice (49). The role of stromal interactions in LM pathology, as indicated by research in animal models, is an interesting area for further exploration. However, additional research is necessary to understand the potential functions of stromal cells in LM progression and to validate the findings through clinical studies.

## Outlook and concluding remarks

Our knowledge of the genetic causes of LMs has increased greatly in recent years. Given that LMs often are caused by some of the same mutations in the PI3K/AKT/mTOR and RAS/MAPK pathways that promote cancer, several drugs already approved for cancer treatment can be repurposed for patients with LM, thus shortening the clinical approval process. Although the inhibitors targeting PI3K and mTOR have shown promising results in suppressing adverse symptoms and improving functional impairments in patients with LM, they are not curative nor effective at regressing established vessel overgrowth in most cases, and they frequently carry serious side effects. The search for additional putative drug targets that are more effective either on their own or in combination with current treatments is therefore of importance. As discussed above, it is emerging from animal studies that paracrine mechanisms acting in synergy with disease-causing oncogenic mutations may play a crucial role in driving LM pathogenesis, thus opening up therapeutic opportunities. For instance, in mice, it is possible to suppress *Pik3ca*-driven lymphatic overgrowth by inhibiting the effects of prolymphangiogenic VEGF-C–producing macrophages through VEGF-C blockade or antiinflammatory COX2 inhibition using celecoxib (Figure 3) (51). Interestingly, in a patient who had undergone unsuccessful surgical intervention and sclerotherapy, the administration of celecoxib was reported to reduce the LM lesion volume without adverse effects (86). Thus, combinatorial treatment of LMs with both rapamycin and VEGF-C/VEGFR3 inhibitors or antiinflammatory drugs might present a promising avenue to pursue in patients with LMs. This may allow the usage of lower doses of the drugs, thus circumventing adverse side effects with improved therapeutic outcomes.

## Figures and Tables

**Table 1:**
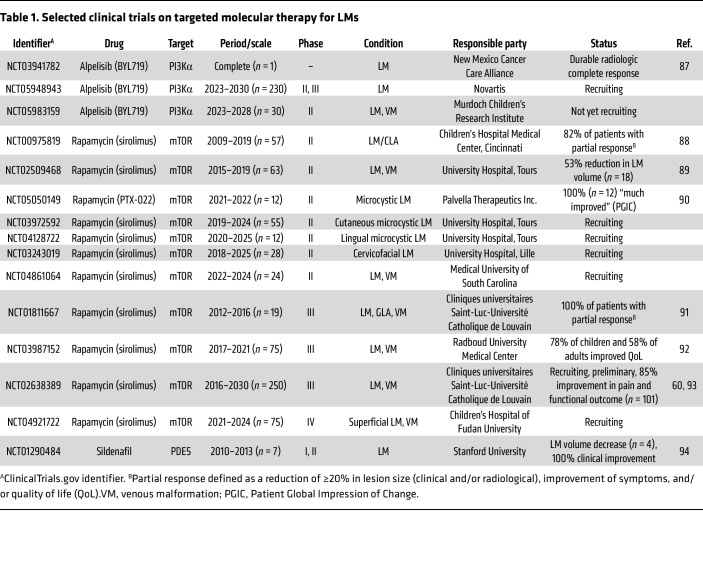
Selected clinical trials on targeted molecular therapy for LMs

## Version 1 03/15/2024

Electronic publication
